# OLDER ADULTS’ PERSPECTIVES OF COGNITIVE HEALTH PROMOTION AND ACTIVITIES TO AMELIORATE COGNITIVE DECLINE

**DOI:** 10.1093/geroni/igz038.1732

**Published:** 2019-11-08

**Authors:** Juanita R Bacsu, Shanthi Johnson, Marc Viger

**Affiliations:** 1 University of Regina, Regina, Saskatchewan, Canada; 2 University of Alberta, Edmonton, Alberta, Canada; 3 University of Saskatchewan, Saskatoon, Saskatchewan, Canada

## Abstract

There is a paucity of research on cognitive health promotion from the perspective of older adults, especially within a rural context. However, dementia and cognitive impairment are more prevalent among rural older adults than urban older adults. This presentation has two objectives: to examine cognitive health promotion from the perspective of rural older adults; and 2) to identify key activities associated with ameliorating cognitive decline in rural communities. Drawing on a community-based participatory research approach, data was collected through two waves of semi-structured interviews with the same group of 42 older adults in rural Saskatchewan, Canada. Guided by lay theory and cultural schema theory, data was coded using thematic analysis. In describing cognitive health promotion, four key areas emerged including emotional health, intellectual health, social health, and functional health. In discussing activities to support cognitive health promotion, participants emphasized the importance of thinking positively, keeping your brain active, mingling with others, and managing your daily affairs. Focusing on older adults’ perceptions of cognitive health promotion provides valuable information to advance knowledge of key strategies and activities to support cognitive health. In developing effective strategies to promote cognitive health, it is essential to engage in collaborative research and partnerships with older adults.

